# AD-VAE: Adversarial Disentangling Variational Autoencoder

**DOI:** 10.3390/s25051574

**Published:** 2025-03-04

**Authors:** Adson Silva, Ricardo Farias

**Affiliations:** Systems Engineering and Computer Science Program (PESC/COPPE/UFRJ), Federal University of Rio de Janeiro, Rio de Janeiro 21941-972, Brazil

**Keywords:** face recognition, GAN, single sample

## Abstract

Face recognition (FR) is a less intrusive biometrics technology with various applications, such as security, surveillance, and access control systems. FR remains challenging, especially when there is only a single image per person as a gallery dataset and when dealing with variations like pose, illumination, and occlusion. Deep learning techniques have shown promising results in recent years using VAE and GAN, with approaches such as patch-VAE, VAE-GAN for 3D Indoor Scene Synthesis, and hybrid VAE-GAN models. However, in Single Sample Per Person Face Recognition (SSPP FR), the challenge of learning robust and discriminative features that preserve the subject’s identity persists. To address these issues, we propose a novel framework called AD-VAE, specifically for SSPP FR, using a combination of variational autoencoder (VAE) and Generative Adversarial Network (GAN) techniques. The proposed AD-VAE framework is designed to learn how to build representative identity-preserving prototypes from both controlled and wild datasets, effectively handling variations like pose, illumination, and occlusion. The method uses four networks: an encoder and decoder similar to VAE, a generator that receives the encoder output plus noise to generate an identity-preserving prototype, and a discriminator that operates as a multi-task network. AD-VAE outperforms all tested state-of-the-art face recognition techniques, demonstrating its robustness. The proposed framework achieves superior results on four controlled benchmark datasets—AR, E-YaleB, CAS-PEAL, and FERET—with recognition rates of 84.9%, 94.6%, 94.5%, and 96.0%, respectively, and achieves remarkable performance on the uncontrolled LFW dataset, with a recognition rate of 99.6%. The AD-VAE framework shows promising potential for future research and real-world applications.

## 1. Introduction

Active since 1960 [1], the field of automatic face recognition has grown dramatically in the last few years, particularly with the rise of deep learning in recent years [2], becoming one of the most popular biometric methods with a wide range of applications [3]. These applications include security, surveillance, and access control systems. The increase is driven by technological advances (primarily through parallel processing in graphics processing units), new technologies for information processing, and security issues. These advances have awakened the interest of researchers in image processing, neural networks, computer vision, and computer graphics [4]. The main advantage of face recognition is that it does not require user cooperation, making it the least intrusive biometrics method.

Despite these advances, face recognition remains challenging in scenarios where only a single image per person is available for training. This is particularly critical in real-world cases such as criminal investigations, where the only image might come from an identity document or a passport. This problem, known as Single Sample Per Person Face Recognition (SSPP FR), is characterized by the need to recognize subjects across varying poses, expressions, and occlusions despite having only one reference image. The lack of sufficient training data makes this problem both relevant and challenging.

SSPP methods have certain advantages, such as reduced dataset complexity and lower computational costs. However, achieving robust and discriminative identity features from a single image remains difficult. Various methods have been proposed to address this challenge, including generic datasets to learn variations (e.g., SSRC [5]), patch-based approaches like DMMA [6], and deep learning models such as VD-GAN [7].

Considering the advances in the use of VAE and GAN, like VAE-GAN for 3D Indoor Scene Synthesis of [8], and the hybrid VAE-GAN of [9] and Patch VAE-GAN of [10], we propose an Adversarial Disentangling Variational Autoencoder (AD-VAE). Specifically designed to address the challenges of SSPP FR by learning robust identity representations, the framework architecture consists of four networks. The first two networks act as a variational autoencoder (VAE) and the other two as Generative Adversarial Networks (GANs) to generate a prototype that conserves the representative identity features of the subject. The VAE part trains the encoder to learn the input image’s distribution of latent space by the variational encoder–decoder process. The GAN part uses a latent code *c* derived from the encoder’s distribution, combined with a Gaussian noise vector to generate an identity-preserving and realistic prototype from the input image *x*. The discriminator of the GAN part works as a multi-task network with three tasks: (1) distinguishing the identity of the input image *x*, (2) distinguishing if the input image contains variations, and (3) determining whether the input image *x* is real or fake. These components work together to achieve better accuracy rates in SSPP FR.

Our method objective is to create an identity-preserver prototype $\hat{x}$ from an input image *x* such that $\hat{x}$ compared to any prototype image with the same identity *x* is close enough using K-Nearest Neighbors (KNN) to be recognized as the same subject. Achieving this objective, we outperform the state-of-the-art SSPP FR methods on four controlled datasets and a wild dataset, LFW. The contributions of this work can be summarized as follows:

- We propose a novel framework, AD-VAE, that combines the ability of VAE to disentangle identity representations with the capacity of the GAN to generate identity-preserving prototypes.
- The AD-VAE achieves state-of-the-art results on four controlled datasets (AR, E-YaleB, CAS-PEAL, and FERET) and the uncontrolled dataset LFW, demonstrating its robustness in handling variations in pose, illumination, and occlusion.
- Unlike other methods, AD-VAE accomplishes this without requiring external pre-trained encoders, making it a self-contained solution for SSPP FR.

The paper is organized into the following sections: Section 2 provides an overview of the related works, as well as a short review of GAN, VAE, and VD-GAN, and shows the proposed method; Section 3 describes the experiments performed on five of the most used public datasets; and Section 4 concludes the study.

## 2. Materials and Methods

The Materials and Methods section describes with sufficient detail the proposed AD-VAE method which integrates the VAE disentangled representation learning and GAN synthesis fidelity. The section is divided into Section 2.1, related works, Section 2.2, the background of the method, and Section 2.3, the description of the proposed method.

### 2.1. Related Works

Throughout the last few decades, various SSPP FR methods have arisen, as well as the classifications forms. Observing the methods and classifications reviewed, we can propose a classification in two classes: (1) geometric-based methods (most old methods that use eye sizes, distances, etc.); (2) appearance-based methods, which are sub-divided into (1) holistic, that uses the whole face as the method input, (2) local, that use patches of the face, with or without superposing/superposition, thus using local features and relationships; and (3) hybrid, using the junction of cited sub-classes (1) and (2). Using this classification, we classify our method as the holistic appearance-based method.

From the reviewed methods for SSPP FR, most of them use a generic dataset to learn about face variations, being holistic-based or patch-based, like a S^3^RC [11] or VD-GAN [7]. The generic dataset is a face database with variations like pose, occlusion expressions, and illumination. The subject’s identities in the generic database must not be in the enrollment database. Some methods use image synthesis to supply the lack of images for training in the enrollment dataset, such as [12]. Other authors like [13] use patch-based methods that divide a face image into small parts and use these parts for recognition. For instance, 3D face reconstruction and illumination transfer techniques have been employed to enhance reference datasets [14]. Similarly, the Uniform Generic Representation (UGR) method combines local and global generic representations to handle variations in pose, illumination, and occlusion [15].

Most of the reviewed methods are based on comprehensive sensing [16], like Sparse Representation Classifier (SRC) or Collaborative Representation Classifier (CRC). Highlighting the SSRC [5] as the principal representative, considering that a sample image can be reconstructed as a linear combination of all training samples and classified leads to the minimal residual. All SSRC processes are guided by Equation (1). Various methods use variations of Equation (1) and an auxiliary generic dataset to complement the lack of information about variations in SSPP enrollment sets:

(1) $$\begin{array}{c} y=P\alpha +V\beta +z \end{array}$$

where P is the sample dictionary, V is the variation dictionary, *z* is the noise error, α is the sparse coefficient vector that selects a few samples from the P dictionary, and β is the sparse coefficient vector that selects a small subset from the variation dictionary V.

In our review, methods based on machine learning have achieved the best results, like the CJR-RACF [17] and VD-GAN [7]. Both techniques use deep convolutional networks. The CJR-RACF uses a patch-based convolutional network. The method in [17] divides the input image into local regions and learns local and global discriminative features. The VD-GAN generates an identity-preserving prototype of the input image with a representative identity feature by convolutional networks. Hybrid approaches such as the combining of hand-crafted features with deep learning features from CNNs have also shown promise in improving recognition accuracy [18].

VD-GAN is based on the image synthesis method DR-GAN [19] that uses GAN [20] combined with a disentangling approach to generate realistic images. Other image synthesis methods, such as AVAE [21], ID-GAN [22], InfoGAN [23], and DisP+V [24], can serve as a foundation for creating SSPP FR methods by leveraging the combined strengths of VAE [25] and GAN. Specifically, VAE contributes by learning a continuous latent space that captures identity-preserving features while being robust to variations in pose, lighting, and occlusion, which is essential for face recognition. GAN complements this by generating high-quality images that maintain identity consistency, with its discriminator enabling multi-task learning to distinguish between real and synthetic images, identify the subject’s identity, and detect variations. This work builds on the AVAE framework and the identity disentangling of the VD-GAN framework, taking advantage of robust feature representation of VAE and the capacity of GAN to generate realistic, identity-preserving prototypes.

### 2.2. Background

#### 2.2.1. Generative Adversarial Networks

Initially created by [20] to generate synthetic images, the GAN was used to solve various problems, like face recognition. This method consists in two networks that play a minimax two-player game. The first network is the generator G, which uses a random noise vector **z** (sampled from a random Gaussian distribution) to generate an image that aims to look like a real one. The second network is Discriminator *D*, which determines if an input image is real or fake. The game consists of generator *G* generating an image that looks real enough to fool discriminator *D*, and discriminator *D* tries not to be fooled by generator *G*. In practice, to provide stronger gradients, the *G* can be trained by maximizing logD(G(z)). This adversarial game between Generator *G* and Discriminator *D*, where each strives to outsmart the other, is represented by the value function V(G,D):

(2) $$\begin{array}{c} \max_{D}V_{D}\left(G,D\right)=E_{x∼p_{\mathrm{data}}\left(x\right)}\left[\log D(x)\right]\;\;\\ +E_{z∼p_{z}\left(z\right)}\left[\log\left(1-D(G(z))\right)\right]\;\; \end{array}$$

(3) $$\max_{G}V_{G}\left(G,D\right)=E_{z∼p_{z}\left(z\right)}\left[\log\left(D(G(z))\right)\right]$$

where $p_{\mathrm{data}}$ is the distribution of training images, and $p_{z}$ is a distribution of noise.

#### 2.2.2. Variational Autoencoders

Proposed by [26], the variational autoencoder (VAE) assumes that an image *x* of training data *X* is the result from a deterministic function f(z) in a random variable z∼p(z) in latent space *Z* such as f:(z,ϵ)→x, ϵ being a stochastic noise. The probability of observing *z* knowing *x* is estimated by a *decoder* $p_{\theta_{d}}:z\mapsto p_{\theta_{d}}\left(x|z\right)$ parameterized by $\theta_{d}$, and the probability that *z* is the latent source of *x* is estimated by *encoder* $q_{\theta_{e}}:x\mapsto q_{\theta_{e}}\left(z|x\right)$ parameterized by $\theta_{d}$. Being that the data $X=(x^{\left(1\right)},...,x^{\left(n\right)})$ with *n* is the number of samples in the data, the parameters of the model are obtained by maximizing the log-likelihood of the observations: $\log p_{\theta_{d}}\left(x^{\left(i\right)}\right)=\log\int_{Z}p_{\theta_{d}}\left(x^{\left(i\right)}|z\right)p\left(z\right)dz$. The $\log p_{\theta_{d}}\left(x^{\left(i\right)}\right)$ is computed by maximizing a tractable lower bound, training the VAE with the following loss function:

(4) $$\begin{array}{cc} L_{VAE}\left(\theta_{e},\theta_{d};x\right)) & =E_{q_{\theta_{e}}\left(z|x\right)}\left[-\log p_{\theta_{d}}\left(x|z\right)\right]\\ & +KL(q_{\theta_{e}}\left(z|x\right)p\left(z\right)))\;\; \end{array}$$

where $p_{\theta_{d}}$ is usually chosen as a Gaussian distribution $N(x;\mu_{\theta_{d}}\left(z\right),Id)$ and KL is the Kullback–Leibler divergence [10,27]. This Equation (4) estimates the reconstruction error and forces the distribution of the latent space to match with p(z). The standard Gaussian distribution, N(z;0,Id), is typically chosen for p(z) due to its simplicity and flexibility.

Equation (4) uses the KL divergence to measure the difference between the approximate distribution of the latent space, $q_{\theta_{e}}\left(z|x\right)$, obtained by the encoder, and the prior distribution p(z). Minimizing KL divergence ensures that the distribution of the latent space is as close as possible to the prior distribution, preventing the encoder from learning a complex distribution or one that is not useful for generating new data.

#### 2.2.3. Variation Disentangling Generative Adversarial Networks

The Variation Disentangling Generative Adversarial Network (VD-GAN) proposed by [7] uses a structure based on GAN, with a generator *G* and discriminator *D*. The generator *G* in VD-GAN consists of two networks, $G_{enc}$ and $G_{dec}$, like an autoencoder. The $G_{enc}$ network receives an image *x* from training data *X*, and aims to learn an identity representation $f\left(x\right)=G_{enc}\left(x\right)$, while the network $G_{dec}$ aims to synthesize a prototype image $\hat{x}$ with the same subject identity of *x* by using the learned identity f(x) and a random noise vector z∼p(z) (from a Gaussian distribution) $\hat{x}=G_{dec}\left(f\left(x\right),z\right)$.

The discriminator *D* is used like a multi-task network with three sub-discriminators $D^{id}$, $D^{var}$, and $D^{gan}$. The sub-discriminator $D^{id}$ is used like a classifier to define the input image identity. The network output is an $N_{d}$-dimensional vector, with $N_{d}$ being the total number of identities. The second task $D^{var}$ is a binary classifier to distinguish if an input image has a variation (any image different from the real prototype is considered with variation). The third task is a classic GAN task, where $D^{gan}$ distinguishes if the generated image is real or fake.

The method training the autoencoder *G* has the following objective function:

(5) $$\begin{array}{c} \max_{G}V_{G}=V_{G}^{gan}+\mu_{1}V_{G}^{id}+\mu_{2}V_{G}^{var}-\mu_{3}V_{G}^{rec} \end{array}$$

where $\mu_{1}$, $\mu_{2}$ and $\mu_{3}$ are the weighting hyper-parameters for the hybrid objective $V_{G}$, and $V_{G}^{rec}$ is the reconstruction loss of the prototype. The discriminator is trained with the following objective function:

(6) $$\begin{array}{c} \max_{D}V_{D}=V_{D}^{gan}+\lambda_{1}V_{D}^{id}+\lambda_{2}V_{D}^{var} \end{array}$$

where $\lambda_{1}$ and $\lambda_{2}$ are trade-off parameters.

### 2.3. The Proposed Method

**Figure 1:**
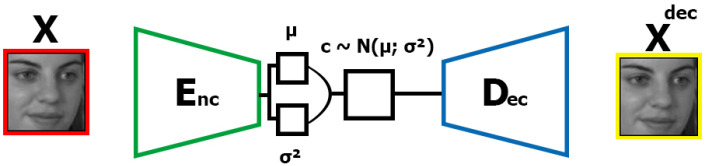
The first part of the proposed AD-VAE, which works as a variational adversarial autoencoder. The x denotes the image data from X, and $x^{dec}$ denotes the decoder reconstruction from x. The encoder $E_{nc}$ has as input image x and produces two outputs, the mean (μ) and the log-variance (σ), which define the parameters of a normal distribution N(μ,σ). From distribution N(μ,σ), we extract a latent vector c∼N(μ,σ) that serves as input to decoder $D_{ec}$ which outputs the reconstruction $x^{dec}$.

**Figure 2:**
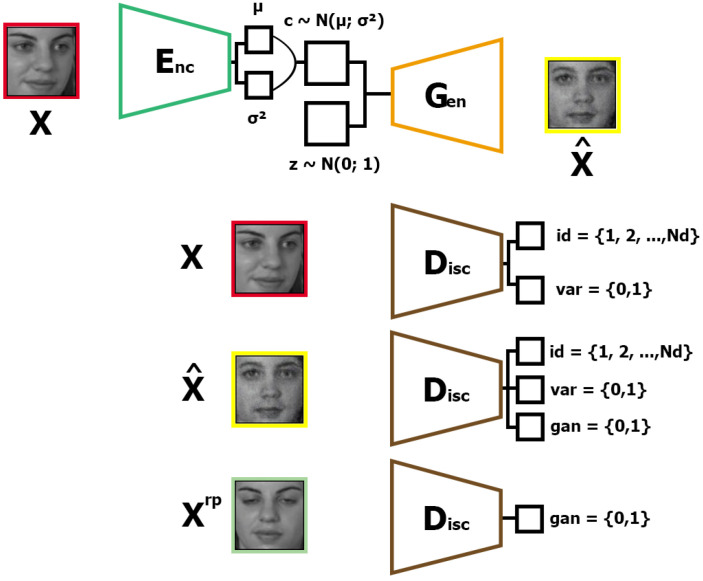
The second part of the proposed architecture of AD-VAE, where x denotes the image from SSPP data X, $x^{rp}$ denotes the image real prototype x, and $\hat{x}$ is the generated prototype from image x. The pre-trained (first part) encoder $E_{nc}$ generates the mean μ and variation σ of x. From distribution N(μ,σ), we extract a latent vector c∼N(μ,σ) that concatenates with noise vector z∼N(0,1) to serve as the input to generator $G_{en}$ which outputs the prototype $\hat{x}$ of x. The discriminator D (1) determines the id and variation of x; (2) determines the id, variation, and whether $\hat{x}$ is real or fake; and (3) determines whether $x_{rp}$ is real or fake.

The proposed method AD-VAE combines the effective disentangled representation learning of the VAE-based approach described by [23] and the high-fidelity synthesis of the GAN-based techniques described by [22]. Similar to [7], we use a variation disentangling and discriminative identity representation via a GAN-based network but joined with a disentangled representation of the VAE base. The architecture of AD-VAE can be divided into two parts, a VAE part and a GAN part. The first part of the AD-VAE architecture is illustrated in Figure 1 and the second part in Figure 2.

The proposed method is formed by four networks that have the training divided into two parts. All method parts are performed sequentially. The first two networks are similar to the original VAE. Using an encoder $E_{nc}\left(x\right)$ that has as input an image $x∼P_{data}\left(x\right)$ and outputs a mean μ and variance σ from the latent space of *x*, from the distribution formed by outputs mean and variance, vector c∼N(μ,σ) is sampled. This sample vector *c* is the input of $D_{ec}\left(c\right)$ that outputs an image reconstruction $x_{rec}$ of the image *x*. The first part of the method aims to train the encoder to learn more disentangled representation from the image distribution. Like [21], the reconstruction error is incorporated into the ELBO (Evidence Lower Bound), which is estimated using the Kullback–Leibler (KL) divergence between the posterior distribution q(z|x) and the prior distribution p(z). The ELBO is defined as

(7) $$\begin{array}{c} \frac{1}{2}\sum_{j=1}^{dim(Z)}\sigma_{E_{nc}j}^{2}+\mu_{E_{nc}}^{2}{\left(x\right)}_{j}-1-\log\sigma_{E_{nc}j}^{2} \end{array}$$

Then, the objective function of the VAE part is defined as the following:

(8) $$\begin{array}{c} L_{VAE}\left(E_{nc},D_{ec};x\right))=\frac{1}{2}{∥\mu_{D_{ec}}\left(z\right))-x∥}^{2}\;\;\;\\ \;+\frac{1}{2}\sum_{j=1}^{dim(Z)}\sigma_{E_{nc}j}^{2}+\mu_{E_{nc}}^{2}{\left(x\right)}_{j}-1-\log\sigma_{E_{nc}j}^{2} \end{array}$$

After the VAE training, the second part is trained with a frozen encoder $E_{nc}$ and using a structure encode generator, like a normal autoencoder. The generator $G_{en}$ uses as input a sample *c* from an encoder distribution of *x*, $c∼N(\mu_{E_{nc}},\sigma_{E_{nc}})$, and a random noise *z* from a Gaussian distribution z∼N(0,1). Naming the function that extracts a sample from the encoder distribution as $f\left(E_{nc}\left(x\right)\right)=f\left(\mu_{E_{nc}},\sigma_{E_{nc}}\right)=c$, we have that $\hat{x}=G_{en}\left(f\left(E_{nc}\left(x\right)\right),z\right)$, with $\hat{x}$ being the generated image with the same identity as *x*. For training the $G_{en}$, we use the following five objective functions:

(9) $$\begin{array}{c} \max_{G_{en}}V_{G_{en}}=V_{G_{en}}^{gan}+\mu_{1}V_{G_{en}}^{id}+\mu_{2}V_{G_{en}}^{var}+\mu_{3}V_{G_{en}}^{rec}+L_{C} \end{array}$$

where $\mu_{1}$, $\mu_{2}$, and $\mu_{3}$ are the weighting hyper-parameters for $V_{G_{en}}$. The sub-objectives are defined as follows:

(10) $$\begin{array}{c} V_{G_{en}}^{id}\left(G_{en},D^{id},c,z\right)=E_{c,y_{id},z}\left[\log D_{y_{id}}^{id}\left(G\left(c,z\right)\right)\right] \end{array}$$

(11) $$\begin{array}{c} V_{G_{en}}^{var}\left(G_{en},D^{var},c,z\right)=E_{c,y_{var},z}\left[\log D_{y_{var}}^{var}\left(G\left(c,z\right)\right)\right] \end{array}$$

(12) $$\begin{array}{c} V_{G_{en}}^{gan}\left(G_{en},D^{gan},c,z\right)=E_{c,z}\left[\log D^{gan}\left(G_{en}\left(c,z\right)\right)\right] \end{array}$$

(13) $$\begin{array}{c} V_{G_{en}}^{rec}\left(G_{en},x_{rp},z\right)=E_{x_{rp},z}\left[\frac{1}{2}{∥x_{rp}-G_{en}\left(f\left(E_{nc}\left(x_{rp}\right)\right),z\right)∥}^{2}\right] \end{array}$$

(14) $$\begin{array}{c} L_{C}\left(G_{en},x,\hat{x}\right)=\frac{1}{2}{∥\frac{\mu_{E_{nc}}\left(x_{rp}\right)\;-\mu_{E_{nc}}\left(\hat{x}\right)}{\sigma_{E_{nc}}\left(\hat{x}\right)}∥}^{2} \end{array}$$

where $x,x_{rp},y_{id},y_{var}$ come from the training data $X=\{\left[x^{1},x_{rp}^{1},y_{id}^{1},,y_{var}^{1}\right],...,\left[x^{n},x_{rp}^{n},y_{id}^{n},,y_{var}^{n}\right]\}$. Being that $x^{i}$ is a random image of subject *i*, $x_{rp}^{i}$ is a real prototype image of subject *i*, $y_{id}^{i}$ is the label identity of subject *i*, and $y_{var}^{i}$ is the label that distinguishes whether $x^{i}$ has variation.

The sub-objective functions of generator $G_{en}$ have the following objectives:

- $V_{G_{en}}^{id}$: Enable $D^{id}$ to classify the generated prototype image $\hat{x}$ as the same identity of label $y^{id}$ of x.
- $V_{G_{en}}^{var}$: Enable $D^{var}$ to detect that there are no variations in $\hat{x}$.
- $V_{G_{en}}^{gan}$: Fool $D^{gan}$ to classify the generated prototype $\hat{x}$ as a real prototype.
- $V_{G_{en}}^{rec}$: Enable the generator to generate an image $\hat{x}$ closest to the real prototype image $x_{rp}$.
- $L_{C}$: Enable the generator to generate an image $\hat{x}$ such that the prior distribution of $E_{nc}\left(\hat{x}\right)$ is closest to the prior distribution of $E_{nc}\left(x_{rp}\right)$.

The last network D is trained using an objective function as follows:

(15) $$\begin{array}{c} \max_{D}V_{D}=V_{D}^{gan}+\lambda_{1}V_{D}^{id}+\lambda_{2}V_{D}^{var} \end{array}$$

where $\lambda_{1}$ and $\lambda_{2}$ are trade-off parameters, and $V_{D}^{id}$, $V_{D}^{var}$, and $V_{D}^{gan}$ are defined as follows:

(16) $$\begin{array}{c} V_{D}^{id}\left(D^{id},x\right)=E_{x,y_{id}}\left[\log D_{y_{id}}^{id}\left(x\right)\right] \end{array}$$

(17) $$\begin{array}{c} V_{D}^{var}\left(D^{var},x\right)=E_{x,y_{var}}\left[\log D_{y_{var}}^{var}\left(x\right)\right] \end{array}$$

(18) $$\begin{array}{c} V_{D}^{gan}\left(D^{id},x\right)=E_{x_{rp}}\left[\log D^{gan}\left(x_{rp}\right)\right]+\\ E_{x,z}\left[\log\left(1-D^{gan}\left(G_{en}\left(f\left(E_{nc}\left(x\right)\right),z\right)\right)\right)\right] \end{array}$$

The discriminator D has the following objectives:

- $V_{D}^{id}$: Predict a correct identity of input image x as labeled in $y_{id}$.
- $V_{D}^{var}$: Predict a correct occurrence of variation on input image x as labeled in $y_{var}$.
- $V_{D}^{gan}$: Predict the real prototype image $x_{rp}$ as real and predict a generate prototype image $\hat{x}$ as fake.

The four networks are training sequentially, with $E_{nc}$ and $D_{ec}$ trained by Equation (8), $G_{en}$ trained by Equation (9), and D by Equation (15). As a result, the encoder $E_{nc}$ learns a latent space with more representative identity from image x by VAE-based method, and the generator $G_{en}$ learns to create a prototype image $\hat{x}$ preserving more identity features of the subject from x. The following section demonstrates the effectiveness of the proposed method.

## 3. Results

In this section, we present a dataset setup and the methodology for the experiments, including the implementation details, and the method effectiveness by experimental results.

### 3.1. Data Collection, Pre-Processing, and Feature Selection

For the tests, we use five widely recognized databases in face recognition, selected for their frequent use in reviewed papers and based on their variability in pose, illumination, occlusion, and real-world conditions. The datasets contain a mix of controlled and uncontrolled environments to ensure diversity and robustness in evaluation. The datasets are the following:

- **AR** [28] consists of 126 identities, having 26 images with expression, illumination, and conclusion per subject. From this dataset, we use a subset with 100 identities. Randomly, we choose 50 identities for the training set and 50 for the test set.
- **Extend Yale B (E-YaleB)** [29] consists of 38 identities under a wide range of lighting conditions, including variations in light intensity (ranging from low to high), different types of lighting (such as natural light, artificial light, and directional lighting) and various light angles (e.g., frontal, lateral, and top–down). Due to the low number of subjects, according to [7], we introduce the AR lighting subset into E-YaleB to extend the number of identities. We randomly choose 100 identities from the mixed dataset for the training set and the remaining 38 identities for the test set.
- **FERET** [30] consists of 1199 identities with variations in gender, age, and ethnicity. From this dataset, we use a subset of 200 identities containing only four pose variations. We randomly choose 150 identities for the training set and the remaining 50 for the test set.
- **CAS-PEAL** [31] consists of 1040 identities with variations like poses, occlusions, and ages. From this dataset, we use a subset with 300 identities from normal and accessory categories, with a neutral image and another 6 wearing different glasses and hats. We randomly choose 200 identities for the training set and the remaining 100 for the test set.
- **LFW** [32] consists of 5749 identities collected under an uncontrolled environment, with a wide range of expressions, poses, illuminations, and other variations. We use a subset of 158 identities with more than ten images per subject from the aligned version of LFW, the LFW-a. For evaluation, we choose 50 identities containing neutral face images for the test set and the other 108 for the training set.

According to [2], the AR and LFW datasets exhibit more complex facial variations compared to others, making them particularly challenging for SSPP FR.

We apply some pre-processing steps on the data to ensure the consistency across all datasets:

- **Image resizing**: All images are resized to 64x64 pixels to match the input dimensions of the network.
- **Normalization**: Pixel values are normalized to the range [0, 1] to improve convergence during training.
- **Alignment**: For the LFW dataset, we use the aligned version (LFW-a) to reduce variations caused by misalignment.
- **Handling missing values:** All datasets used in this study contain complete data, with no missing values, eliminating the need for further data imputation.

The networks process the pre-processed data as follows:

- **Latent code generation:** The encoder ($E_{nc}$) generates the mean (μ) and variance (σ) of the latent space distribution. The latent code (*c*) is then sampled from this distribution using the reparameterization trick, which ensures that *c* is differentiable with respect to the network’s parameters. This differentiability allows for gradient-based optimization during training. A noise vector (*z*) is sampled independently from a Gaussian distribution for variation modeling.
- **Feature concatenation:** The generator ($G_{en}$) combines *c* and *z* into a single input vector (c,z) to create identity-preserving prototypes with controlled variations.
- **Representation dimensionality:** For all datasets, the latent dimension ($L_{dim}$) is set to 100, ensuring consistent feature representation across datasets.

The AD-VAE networks’ architectures are shown in Table 1 and Table 2. The networks $E_{nc}$ and D are similar, differing only in the final layer, having $E_{nc}$ two fully connected layers, one layer that outputs a mean μ and another to the variance σ, both with dimension $L_{dim}$. For network D, the last layer is a fully connected layer with output dimension $N_{dim}+2$, where $N_{dim}$ is the number of identities ($D^{id}$) of the training set. The other two positions (+2) are used to distinguish the occurrence of variation ($D^{var}$) and if the input image is real or fake ($D^{gan}$).

The networks $G_{en}$ and $D_{ec}$ are also similar, with a difference in the top layer. The first layer of $G_{en}$ uses an input of dimension $L_{dim}*2$ because the input is a concatenation of latent code *c* and noise vector *z*. The $D_{ec}$ first layer uses an input of dimension $L_{dim}$ because only a latent code *c* is used as input.

The network’s weights are initialized from a zero-centered Gaussian distribution with a standard deviation of 0.002. Training is performed using mini-batch stochastic gradient descent with a batch size of 16. The Adam optimizer [33] is employed with the following learning rates for each network: 0.0002 ($E_{nc}$), 0.0002 ($D_{ec}$), 0.0001 ($G_{en}$), and 0.0003 (D). For all datasets, we set the parameters $\mu_{1}$, $\mu_{2}$, $\mu_{3}$ in Equation (9) and $\lambda_{1}$, $\lambda_{2}$ in Equation (15) as 2.0, 0.5, 0.1, 2.0, and 0.5, respectively, as used by [7].

### 3.2. Evaluation in Single Sample Face Recognition

**Figure 3:**
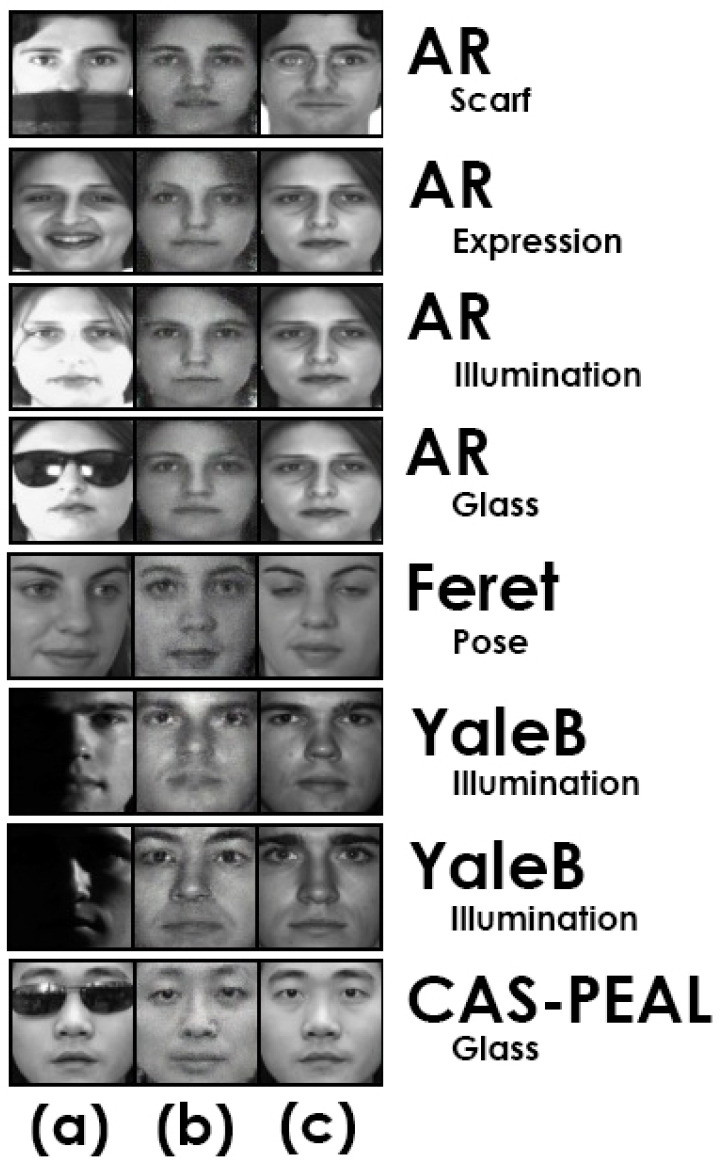
The prototypes generated by AD-VAE are presented as follows: (**a**) the sample image with variations, (**b**) the generated prototype of image (**a**), and (**c**) the real prototype of image (**a**). On the right side, the name of the dataset and the variation are indicated.

This section describes the results of the evaluation of AD-VAE on the task of recognizing a face that has only a near-neutral face as an enrollment sample. Initially, the experiment is carried out on controlled datasets AR, CAS-PEAL, FERET, and E-YaleB, and is finally implemented on the wild dataset LFW. We define the methods and dataset configurations as in [7]. Figure 3 shows samples of dataset images, showing the test image x, the prototype of the test image $\hat{x}$, and the real prototype of the test image $x_{rp}$.

For comparison in the controlled datasets, we use nine methods of the literature: PCA [34], VAE [25], SRC [35], CRC [36], PCRC [37], DMMA [6], SVDL [38], SLRC [39], and S^3^RC [11]. The training set works as the generic set for methods that use a generic gallery set to learn the variations of faces.

For DMMA and PCRC, the patch is non-overlapped with a patch size of 16x16 pixels. The other parameters of DMMA are set as $K_{1}=30,K_{2}=2,K=2$, and σ=10. We fix the regularization parameter λ of SRC, CRC, SLRC, and S^3^RC as 0.01. For SVDL, the parameters are set as $\lambda_{1}=0.001,\lambda_{2}=0.01$, and $\lambda_{3}=0.0001$. To measure the similarity of generated prototypes in PCA and VAE, we use the cosine distance metric and KNN with k=1 as a classifier. For VD-GAN and AD-VAE, we generate a prototype of query image *y* and a prototype of each $x_{rp}$. Then, we use the KNN with a cosine distance to classify the generated prototypes of the same subject. Considering P(x) as a generated prototype from *x* in Equation (19), we classify the generated prototypes by Equation (20):

(19) $$\begin{array}{c} P\left(x\right)=G_{en}\left(f\left(E_{nc}\left(x\right)\right),z\right) \end{array}$$

(20) $$\begin{array}{c} ID\left(y\right)=\underset{k}{\arg\min}\;dist\left(P\left(y\right),P\left(x_{rp}\right)\right) \end{array}$$

Table 3 presents the recognition accuracy on four controlled datasets. Results show that the AD-VAE outperforms all other methods on four datasets. Our method achieves accuracy rates superior to VD-GAN, which overcomes the generic data learning methods. Like the VD-GAN, the proposed method outperforms the linear-based superposition model methods concerning non-linear variations, as pose variation in the FERET dataset. The AD-VAE overcomes the others due to the learning variation being disentangled over the encoder training, resulting in a latent vector more representative of each identity.

Even with similarities, the encoder training by VAE and the additional $L_{C}$ loss function could outperform VD-GAN. According to the authors of VD-GAN, the VAE is not competitive due to being an unsupervised method, whereas the AD-VAE uses the strengths of the VAE in a supervised form, thus achieving better rates than VD-GAN.

To test in the wild dataset, we evaluate the AD-VAE, comparing with VD-GAN_*Lcnn*_ (VD-GAN version using LightCNN-29 as the encoder in autoencoder Generator G) and four recent deep learning-based methods: JCR-ACF [40], Regular-face [41], Arc-face [42], and CJR-RACF [17]. Table 4 reports the rank-1 recognition rates of all the methods for SSPP FR, showing that our AD-VAE outperforms all the others, even the VD-GAN with encoder modifications.

## 4. Conclusions

We proposed the AD-VAE framework, which, to the best of our knowledge, is the first to leverage a variational autoencoder in the Single Sample Per Person Face Recognition (SSPP FR) problem. The key contributions of this research include the development of a novel method capable of learning identity-preserving prototypes from both controlled and wild SSPP FR datasets. The standard form of AD-VAE outperformed all SSPP FR techniques tested without any alteration of the AD-VAE original model, showing the robustness of our approach. AD-VAE effectively deals with huge variations, such as the pose of FERET, illumination, and occlusion of AR, the complex illumination condition of EYaleB, and the mixed variations of the wild dataset LFW.

The results highlight the effectiveness of AD-VAE in handling significant variations, such as pose variations in FERET, illumination and occlusion in AR, the challenging lighting conditions of E-YaleB, and the mixed variations present in the wild dataset LFW. These findings confirm the potential of AD-VAE to address critical challenges in SSPP FR and establish it as a strong candidate for practical applications in real-world scenarios. Additionally, the results demonstrate that variational autoencoders can be effectively used to disentangle identity and variation features in SSPP FR tasks, opening new possibilities for exploring disentangled representations in biometric applications, and the framework achieves high recognition rates without relying on external pre-trained networks, providing a cost-effective and efficient approach to SSPP FR.

However, there are several areas for further improvement and exploration. To enhance the learning capacity for representation of our framework, we could incorporate new network architectures. Potential improvements include intermediate latent space combined with adaptive instance normalization used in StyleGAN2 [43], or the application of diffusion models, such as the one used in the Dual Condition Face Generator (DCFace) [44], for style transfer methods. Furthermore, leveraging larger datasets, such as GAN-Control [45], could further improve performance. An additional enhancement could be the adoption of a separate VAE training step, as in the ID-GAN model [22], which allows for more refined control of face generation and increasing recognition accuracy.

## Figures and Tables

**Table 1:** Structure network of $E_{nc}$ and D. Every Conv2d layer is preceded by a BatchNorm2d normalization layer and a LeakyReLU activation layer.

Encoder $E_{nc}$	Discriminator D	
**Layer**	**input/output**	**Filter/Stride/Padding**
**Conv2d-1**	3/64	4x4/2/1
**Conv2d-2**	64/128	4x4/2/1
**Conv2d-3**	128/256	4x4/2/1
**Conv2d-4**	256/512	4x4/2/1
$E_{nc}$ **Finals layers**	**Flatten**	
**Fullconected**-μ	output = $L_{dim}$	**FullConected**-σ
D Final layers	**Flatten**	
**Fullconected**	output = $N_{dim}+2$	

**Table 2:** Structure network of $D_{ec}$ and $G_{en}$. The ConvTranspose2d layers 1, 2, and 3 is preceded by a BatchNorm2d normalization layer and a ReLU activation layer.

Generator $G_{en}$	Decoder $D_{ec}$	
**Fullconected**	$G(L_{dim}*2/8192)$	$D(L_{dim}/8192$)
**Rechape**(512x4x4)>	**BatchNorm2d**>	**ReLU**
**Layer**	**input/output**	**Filter/Stride/Padding**
**ConvTranspose2d-1**	512/256	4x4/2/1
**ConvTranspose2d-2**	256/128	4x4/2/1
**ConvTranspose2d-3**	128/64	4x4/2/1
**ConvTranspose2d-4**	64/3	4x4/2/1
**Tanh**		

**Table 3:** Recognition accuracies (%) and standard errors (%) of different methods on E-YaleB&AR, CAS-PEAL, AR, and FERET datasets for SSPP FR.

Methods	AR	E-YaleB&AR	CAS-PEAL	FERET
PCA	42.4±2.2	58.5±2.3	51.3±1.0	40.5±31
VAE	44.9±1.1	59.9±1.1	51.4±0.9	55.0±2.3
SRC	49.6±2.4	64.0±3.8	62.3±1.4	51.5±2.6
CRC	50.8±4.8	63.5±1.4	69.5±2.7	43.0±4.1
DMMA	51.9±1.9	55.4±1.1	59.2±0.6	57.5±1.2
PCRC	74.1±3.7	80.7±5.4	75.8±0.6	24.0±2.4
SVDL	76.0±0.8	88.1±1.8	78.7±1.2	67.0±1.7
SLRC	76.6±1.8	88.8±2.6	78.2±3.3	68.0±3.8
S^3^RC	77.8±2.6	88.2±1.5	80.3±3.3	73.0±2.1
VD-GAN	79.7±0.8	90.6±2.5	81.2±2.2	90.5±0.8
AD-VAE	84.9±1.5	94.6±1.8	94.5±1.6	96.0±1.0

**Table 4:** Recognition accuracies (%) of different deep learning-based methods on LFW dataset for SSPP FR.

Methods	Recognition Rate (%)
JCR-ACF	86.0%
Regular-face	83.7%
Arc-face	92.3%
CJR-RACF	95.5%
VD-GAN_*Lcnn*_	98.4%
AD-VAE	99.6±1.2%

## Data Availability

All code data can be accessed from https://github.com/lightdi/ADVAE (last accessed on 15 February 2025).

## Abbreviations

The following abbreviations are used in this manuscript:

SSPP	Single Sample Per Person
FR	Face Recognition
ProgPROG-AD-VAE	Progressive Adversarial Disentangling Variational Autoencoder
VAE	Variational Autoencoder
SRC	Sparse Representation Classifier
CRC	Collaborative Representation Classifier
PCA	Principal Component Analysis
CJR-RACF	Class-level joint representation with regional adaptive convolution features
JCR-ACF	Joint and Collaborative Representation with local Adaptive Convolution Feature
DMMA	Discriminative Multi-manifold Analysis
PCRC	Patch Based CRC
SVDL	Sparse Variation Dictionary Learning
SLRC	Superposed Linear Representation Classifier
S3RC	Semi-supervised sparse representation classifier
VD-GAN	Variation Disentangling Generative Adversarial Network

## References

[1] Lahasan B., Lutfi S.L., San-Segundo R. (2017). A survey on techniques to handle face recognition challenges: Occlusion, single sample per subject and expression. Artif. Intell. Rev..

[2] Liu F., Chen D., Wang F., Li Z., Xu F. (2023). Deep learning based single sample face recognition: A survey. Artif. Intell. Rev..

[3] Minaee S., Abdolrashidi A., Su H., Bennamoun M., Zhang D. (2023). Biometrics recognition using deep learning: A survey. Artif. Intell. Rev..

[4] Zhao W., Chellappa R., Phillips P.J., Rosenfeld A. (2003). Face recognition: A literature survey. ACM Comput. Surv..

[5] Deng W., Hu J., Guo J. In Defense of Sparsity Based Face Recognition. Proceedings of the 2013 IEEE Conference on Computer Vision and Pattern Recognition.

[6] Lu J., Tan Y.P., Wang G. (2013). Discriminative Multimanifold Analysis for Face Recognition from a Single Training Sample per Person. IEEE Trans. Pattern Anal. Mach. Intell..

[7] Pang M., Wang B., Cheung Y.m., Chen Y., Wen B. (2021). VD-GAN: A Unified Framework for Joint Prototype and Representation Learning From Contaminated Single Sample per Person. IEEE Trans. Inf. Forensics Secur..

[8] Li S., Li H. (2023). Deep Generative Modeling Based on VAE-GAN for 3D Indoor Scene Synthesis. Int. J. Comput. Games Technol..

[9] Cheng M., Fang F., Pain C., Navon I. (2020). An advanced hybrid deep adversarial autoencoder for parameterized nonlinear fluid flow modelling. Comput. Methods Appl. Mech. Eng..

[10] Mak H.W.L., Han R., Yin H.H.F. (2023). Application of Variational AutoEncoder (VAE) Model and Image Processing Approaches in Game Design. Sensors.

[11] Gao Y., Ma J., Yuille A.L. (2017). Semi-Supervised Sparse Representation Based Classification for Face Recognition with Insufficient Labeled Samples. Trans. Img. Proc..

[12] Deng W., Hu J., Wu Z., Guo J. (2018). From one to many: Pose-Aware Metric Learning for single-sample face recognition. Pattern Recognit..

[13] Gu J., Hu H., Li H., Hu W. Patch-based alignment-free generic sparse representation for pose-robust face recognition. Proceedings of the 2016 IEEE International Conference on Image Processing (ICIP).

[14] Abdelmaksoud M., Nabil E., Farag I., Hameed H.A. (2020). A Novel Neural Network Method for Face Recognition with a Single Sample Per Person. IEEE Access.

[15] Ding Y., Liu F., Tang Z., Zhang T. (2020). Uniform Generic Representation for Single Sample Face Recognition. IEEE Access.

[16] Hu X., Peng S., Wang L., Yang Z., Li Z. (2017). Surveillance video face recognition with single sample per person based on 3D modeling and blurring. Neurocomputing.

[17] Yang M., Wen W., Wang X., Shen L., Gao G. (2020). Adaptive Convolution Local and Global Learning for Class-Level Joint Representation of Facial Recognition with a Single Sample Per Data Subject. IEEE Trans. Inf. Forensics Secur..

[18] Adjabi I. Combining hand-crafted and deep-learning features for single sample face recognition. Proceedings of the 2022 7th International Conference on Image and Signal Processing and their Applications (ISPA).

[19] Tran L., Yin X., Liu X. Disentangled Representation Learning GAN for Pose-Invariant Face Recognition. Proceedings of the 2017 IEEE Conference on Computer Vision and Pattern Recognition (CVPR).

[20] Goodfellow I.J., Pouget-Abadie J., Mirza M., Xu B., Warde-Farley D., Ozair S., Courville A., Bengio Y. (2014). Generative Adversarial Networks. arXiv.

[21] Plumerault A., Borgne H.L., Hudelot C. (2020). AVAE: Adversarial Variational Auto Encoder. arXiv.

[22] Lee W., Kim D., Hong S., Lee H. (2020). High-Fidelity Synthesis with Disentangled Representation. arXiv.

[23] Chen X., Duan Y., Houthooft R., Schulman J., Sutskever I., Abbeel P. (2016). InfoGAN: Interpretable Representation Learning by Information Maximizing Generative Adversarial Nets. arXiv.

[24] Pang M., Wang B., Ye M., Cheung Y.m., Chen Y., Wen B. (2023). DisP+V: A Unified Framework for Disentangling Prototype and Variation From Single Sample per Person. IEEE Trans. Neural Netw. Learn. Syst..

[25] Tran L., Yin X., Liu X. (2019). Representation Learning by Rotating Your Faces. IEEE Trans. Pattern Anal. Mach. Intell..

[26] Kingma D.P., Welling M. (2013). Auto-Encoding Variational Bayes. arXiv.

[27] Gimenez J.R., Zou J. (2022). A Unified f-divergence Framework Generalizing VAE and GAN. arXiv.

[28] Martinez A., Benavente R. (1998). The AR Face Database.

[29] Georghiades A., Belhumeur P., Kriegman D. (2001). From few to many: Illumination cone models for face recognition under variable lighting and pose. IEEE Trans. Pattern Anal. Mach. Intell..

[30] Phillips P., Moon H., Rizvi S., Rauss P. (2000). The FERET evaluation methodology for face-recognition algorithms. IEEE Trans. Pattern Anal. Mach. Intell..

[31] Gao W., Cao B., Shan S., Chen X., Zhou D., Zhang X., Zhao D. (2008). The CAS-PEAL Large-Scale Chinese Face Database and Baseline Evaluations. IEEE Trans. Syst. Man Cybern. Part A Syst. Hum..

[32] Huang G.B., Mattar M., Berg T., Learned-Miller E. (2008). Labeled Faces in the Wild: A Database for Studying Face Recognition in Unconstrained Environments.

[33] Radford A., Metz L., Chintala S. (2015). Unsupervised Representation Learning with Deep Convolutional Generative Adversarial Networks. arXiv.

[34] Turk M., Pentland A. Face recognition using eigenfaces. Proceedings of the 1991 IEEE Computer Society Conference on Computer Vision and Pattern Recognition.

[35] Wright J., Yang A.Y., Ganesh A., Sastry S.S., Ma Y. (2009). Robust Face Recognition via Sparse Representation. IEEE Trans. Pattern Anal. Mach. Intell..

[36] Zhang L., Yang M., Feng X. Sparse representation or collaborative representation: Which helps face recognition?. Proceedings of the 2011 International Conference on Computer Vision.

[37] Zhu P., Zhang L., Hu Q., Shiu S.C.K., Fitzgibbon A., Lazebnik S., Perona P., Sato Y., Schmid C. (2012). Multi-scale Patch Based Collaborative Representation for Face Recognition with Margin Distribution Optimization. Proceedings of the Computer Vision—ECCV 2012.

[38] Yang M., Van L., Zhang L. Sparse Variation Dictionary Learning for Face Recognition with a Single Training Sample per Person. Proceedings of the 2013 IEEE International Conference on Computer Vision.

[39] Deng W., Hu J., Guo J. (2018). Face Recognition via Collaborative Representation: Its Discriminant Nature and Superposed Representation. IEEE Trans. Pattern Anal. Mach. Intell..

[40] Yang M., Wang X., Zeng G., Shen L. (2017). Joint and collaborative representation with local adaptive convolution feature for face recognition with single sample per person. Pattern Recognit..

[41] Zhao K., Xu J., Cheng M.M. RegularFace: Deep Face Recognition via Exclusive Regularization. Proceedings of the 2019 IEEE/CVF Conference on Computer Vision and Pattern Recognition (CVPR).

[42] Deng J., Guo J., Yang J., Xue N., Cotsia I., Zafeiriou S.P. (2021). ArcFace: Additive Angular Margin Loss for Deep Face Recognition. IEEE Trans. Pattern Anal. Mach. Intell..

[43] Karras T., Aittala M., Hellsten J., Laine S., Lehtinen J., Aila T. (2020). Training Generative Adversarial Networks with Limited Data. arXiv.

[44] Kim M., Liu F., Jain A., Liu X. (2023). DCFace: Synthetic Face Generation with Dual Condition Diffusion Model. arXiv.

[45] Shoshan A., Bhonker N., Kviatkovsky I., Medioni G. (2021). GAN-Control: Explicitly Controllable GANs. arXiv.

